# Alcohol-Related Dysfunction in Working-Age Men in Izhevsk, Russia: An Application of Structural Equation Models to Study the Association with Education

**DOI:** 10.1371/journal.pone.0063792

**Published:** 2013-05-08

**Authors:** Sarah Cook, David A. Leon, Nikolay Kiryanov, George B. Ploubidis, Bianca L. De Stavola

**Affiliations:** 1 London School of Hygiene & Tropical Medicine, London, United Kingdom; 2 Izhevsk State Medical Academy, Izhevsk, Russia; California Pacific Medicial Center Research Institute, United States of America

## Abstract

**Background:**

Acute alcohol-related dysfunctional behaviours, such as hangover, are predictive of poor health and mortality. Although much is known about the association of education with alcohol consumption, little is known about its association with these dysfunctional behaviours.

**Methods:**

The study population was 1,705 male drinkers aged 25–54 years resident in the city of Izhevsk, Russia who participated in a cross-sectional survey (2003–6). Structural equation modelling was used to examine the relationships between education, beverage and non-beverage alcohol intake, drinking patterns, and acute alcohol-related dysfunction score among these drinkers.

**Results:**

Dysfunction was related to all other drinking variables, with the strongest predictors being spirit intake, non-beverage alcohol consumption and drinking patterns. There was a strong relationship between education and acute dysfunction which was not explained by adjusting for alcohol intake and drinking patterns (mean adjusted dysfunction score 0.35 SD (95% CI 0.10, 0.61) lower in men with higher versus secondary education).

**Conclusions:**

Although by definition one or more aspects of alcohol consumption should explain the educational differences in alcohol-related dysfunction, detailed information on drinking only partly accounted for the observed patterns. Thus beyond their intrinsic interest, these results illustrate the challenges in constructing statistical models that convincingly identify the pathways that link educational differences to health-related outcomes.

## Introduction

Hazardous alcohol consumption is a major cause of low life expectancy in Russia and an important public health concern particularly in men.[1]–[6] Drinking in Russia is characterised by episodic consumption of very large volumes of ethanol particularly from spirits.[5], [7]–[9] Whilst spirits remain the dominant beverage type, consumption of beer has been increasing especially in younger people. Russian alcohol use also includes a high prevalence of distinctive hazardous drinking behaviours such as zapoi (two or more days of continuous drunkenness where a person is withdrawn from normal social life) and consumption of non-beverage alcohol i.e. manufactured ethanol-based liquids not intended for drinking (e.g. eau de cologne and medicinal tinctures).[1], [10] All-cause mortality in Russia shows a strong inverse gradient with education but relatively little work has been done to understand the factors - including alcohol consumption- that comprise the mechanism underlying this association. [11]–[14] The studies in Russia which have investigated this found that educational level was associated with measures of hazardous drinking including heavy drinking (more than 160 grams of ethanol a week), binge drinking and drinking more than twice a week, and with alcohol-related problems, however results on the association between education and overall alcohol intake have been inconsistent.[15]–[17]

Consumption of alcohol has many negative consequences both chronic and acute. The most immediate i.e. acute consequence of heavy alcohol use is intoxication or drunkenness often closely followed by hangover. Frequency of these acute consequences of heavy drinking can and have been used as proxy measures of episodes of heavy drinking [18]–[22] but are also negative outcomes in themselves. For example hangover is unpleasant and may have negative consequences economically due to lost productivity, absenteeism and work-related accidents as well as increased risk of injury.[23]–[27] These immediate consequences of alcohol consumption may be described as acute behavioural dysfunctions from alcohol.

Chronic consequences of alcohol consumption have also been shown to be closely associated with acute alcohol-related behavioural dysfunction. Frequency of drunkenness has been found to be a strong predictor of social problems, alcohol dependence and alcohol-related harm.[21]Frequency of intoxication, hangover and passing out because of drunkenness have been shown to be strongly predictive of subjective health, alcohol-related hospital admissions and death even after adjustment for average weekly intake of alcohol.[19], [20]

Acute alcohol-related behavioural dysfunction can be seen as on the pathway between alcohol intake (frequency and quantity of alcohol consumed) and more distal outcomes possibly related to alcohol use such as relationship breakdown or unemployment. Therefore a good measure of acute alcohol-related dysfunction could be a useful tool for understanding the relationship between alcohol consumption and alcohol-related problems and as a predictor of more distal adverse outcomes due to alcohol. Beyond this, these acute dysfunctions could also be important indicators of a pattern of drinking that has serious health consequences. Since hazardous drinking in Russia shows associations with educational level [15], [16] it is likely that there are also educational gradients in dysfunction but this has not previously been investigated.

The aims of the analyses reported here were 1) to investigate what aspects of alcohol consumption (alcohol intake and drinking pattern) are most strongly associated with acute alcohol-related behavioural dysfunctions, and 2) to investigate the relationship between educational level and acute alcohol-related dysfunction and the relative contribution of different aspects of alcohol consumption in explaining this relationship among drinkers.

## Materials and Methods

### Study sample

This study used data from the Izhevsk Family Study 1 (IFS-1). This study included a cross-sectional survey conducted between 2003 and 2006 of 1941 men aged 25–54. These men were a random sample selected from the 2002 population register of the city of Izhevsk. Most of these men had originally been selected as live controls in a case-control study of the relationship between hazardous drinking and premature mortality [1] which involved them being frequency matched by age to cases (deceased men aged 25–54 years resident in Izhevsk). This paper focused only on the live men who had consumed alcohol in the past year (1,705/1,941 men) since only drinkers can be at risk of acute alcohol-related dysfunction.

### Outcome variables

The outcome of interest was self-reported acute alcohol-related behavioural dysfunction in the previous year and was defined in terms of either: (i) *routine acute dysfunction:* measured as a latent variable manifested by self-reported behaviours following alcohol consumption. These were: frequency of excessive drunkenness (*peripivayet*– to get completely drunk), hangover, sleeping in clothes because of drunkenness, and failing to fulfil family or personal obligations because of drinking alcohol. There were seven response categories for these questions: never or almost never, less than once a month, once a month, several times a month, once a week, several times a week, and every day, or (ii) *sporadic acute dysfunction*: at least one episode of *zapoi* (defined as a period of continuous drunkenness of several days or more during which a person does not work and is withdrawn from normal life).Zapoi was considered a dysfunctional behaviour because of the negative impact it can have on an individual's life.[28]

### Exposure variables

Self-reported beverage and non-beverage alcohol intake and drinking patterns in the year preceding the interview were the main exposure variables in the first analyses and educational level in the second.

Beverage alcohol intake was quantified from questions on frequency of beer, wine, and spirit consumption, and on their usual and maximum quantity per drinking occasion (in explicit categories used by Russians in everyday life: beer in bottles and wine and spirits in grams). It was defined in terms of three latent factors representing beer, wine, and spirit intake. The available information was obtained from questions on frequency of consumption (with 7 categories: never or almost never, a few times per year, 1–3 times a month, once or twice a week, three or four times a week, nearly every day, and every day or more often) and questions on usual and maximum quantity (converted into litres of pure ethanol consumed per occasion using the mid-point of each category so that the same unit of measurement was used for beer, wine, and spirits). More specifically the three latent factor scores for beverage alcohol intake were each defined in terms of: intake by usual volume of ethanol consumed, maximum volume of ethanol consumed, and frequency of consumption. Self-reported consumption of non-beverage alcohol in the past year (e.g. eau de cologne) was coded as a binary variable: yes or no.

The information on drinking patterns was derived from questions on whether: (i) men ever drank large quantities of spirits without also eating some food at the same sitting (coded as never, sometimes or always); (ii) they ever drank alone (coded as never, sometimes, or often), and (iii) whether they ever drank before noon (coded as never, occasionally, and frequently). The three indicators of drinking pattern were not highly correlated and therefore were not taken to be manifestations of a common latent dimension.

Educational level was the exposure for the second aim. It was coded in three categories: incomplete secondary or lower, secondary, and higher or incomplete higher.

### Statistical analyses

**Figure 1:**
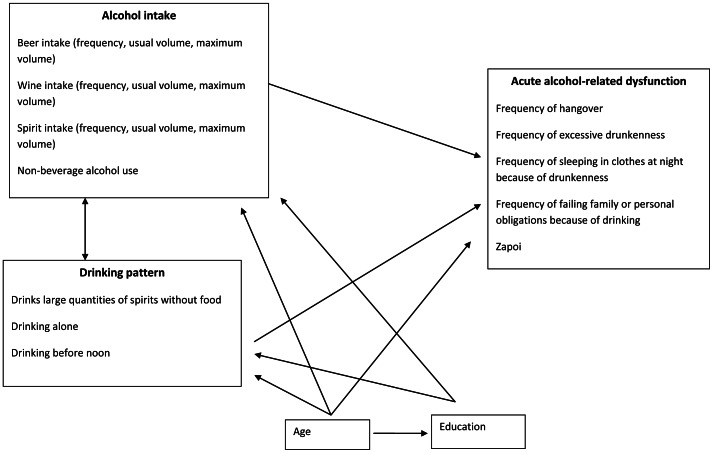
Hypothesized Relationships between Variables in the Izhevsk Family Study 1.

Structural equation models were used to study the association among these variables and each outcome (routine and sporadic acute behavioural dysfunction), according to the conceptual model shown in Figure 1. This approach to modelling has several advantages in particular with the inclusion of latent variables that allow extraction of essential information from the raw data and reduce measurement error, naturally under some distributional and functional assumptions.

Distinct structural equation models for either routine or sporadic acute dysfunction were fitted to address the two aims. All included adjustment for age and measurement models for the observed alcohol intake and dysfunction variables used for the specification of latent variables [29]. To investigate the first aim, i.e. what aspects of alcohol intake were most strongly associated with acute alcohol-related behavioural dysfunction, we fitted models which included only the outcome (either routine or sporadic dysfunction), the three latent alcohol intake variables, non-beverage alcohol use and the indicators of drinking pattern. The linearity of the effects of the latent alcohol variables was examined by including quadratic terms in the model and testing for their significance using the Wald test [30]. Deviance residual were calculated for each of the linear regression models to assess distributional assumptions and to identify outliers. In general linear model assumptions were found to be satisfied. We also checked the specification of the logistic regression models by saving predicted values and refitting each model on these predictions and their squared values. A significant effect of the quadratic term would indicate that the link function is inappropriate however we found no evidence that this was the case in our data.

The selected final specification of the model was then expanded to include education in order to address the second aim. Our conceptual model (Figure 1) does not include a direct relationship between education and acute alcohol-related behavioural dysfunction because associations between education and dysfunction were assumed necessarily to be mediated by alcohol intake and/or patterns of drinking (since alcohol must first be consumed in order to experience its acute consequences). To assess this hypothesis we compared the results obtained from fitting the model corresponding to Figure 1 with one that included a direct relationship between education and dysfunction. This was done separately for routine and sporadic dysfunction. To explain the results we also investigated what aspects of alcohol consumption best explained the relationship between education and alcohol-related dysfunction by sequentially removing each of the drinking variables from the model that included a direct effect of education on acute dysfunction. The reason for this approach was that removing variables lying on the pathway from education to dysfunction should result in an increased effect of education, with the most influential variables leading to the greatest changes.

Effects of categorical explanatory variables were assessed using linear tests for trend based on the Wald test.[30] Interactions between the effects of age, alcohol intake, and drinking patterns with that of education were considered by fitting models stratified by education and assessing improvements in goodness of fit.

Estimation was by Weighted Least Squares with mean and variance adjusted (WMSLV) which is appropriate for the categorical nature of the outcome variables. Model fit was assessed using the Comparative Fit Index (CFI), the Tucker Lewis Index (TLI), and the Root Mean Square Error of Approximation (RMSEA). CFI and TLI values greater than 0.95 indicate good model fit with a minimum of 0.90 indicating acceptable fit.[31], [32] For the RMSEA values greater than 0.10 indicates a bad fit, while less than 0.08 indicates a reasonable fit and values less than 0.05 indicate a good fit.[32]

### Missing data

There was a small amount of missing data for most of the questions on alcohol with the largest amount of missing data affecting the question on the failure to fulfil family or personal obligations (Table 1). The estimation procedure WLSMV allowed the inclusion of incomplete records which would not bias the estimates on the assumption that data were missing completely at random [33]. Comparative analyses were carried out restricting the data to men with complete data for all variables.

**Table 1:** Distribution of Self-Reported Alcohol Intake and Indicators of Acute Alcohol-related Dysfunction in Men who had Consumed Alcohol in the Past 12 Months; Izhevsk Family Study 1.

		N	(% or SD)
Frequency of drinking beer	Never drinks beer	269	(15.8)
(Missing = 1)	A few times per year	148	(8.7)
	1–3 times per month	434	(25.5)
	1–2 times/week	578	(33.9)
	3–4 times/week	164	(9.6)
	Almost daily	90	(5.3)
	Daily	21	(1.2)
Mean usual volume of ethanol per occasion from beer in beer drinkers (mls of ethanol) (Missing = 8)		44.5	(25.8)
Mean maximum volume of ethanol per occasion from beer in beer drinkers (mls of ethanol)(Missing = 20)		74.3	(42.2)
Frequency of drinking wine	Never drinks wine	1047	(61.4)
(Missing = 6)	A few times per year	347	(20.4)
	1–3 times per month	205	(12.0)
	1–2 times/week	84	(4.9)
	3–4 times/week	16	(0.9)
	Almost daily	6	(0.4)
	Daily	0	(0.0)
Mean usual volume of ethanol per occasion from wine in wine drinkers (mls of ethanol) (Missing = 6)		46.2	(33.4)
Mean maximum volume of ethanol per occasion from wine in wine drinkers (mls of ethanol)(Missing = 12)		73.1	(46.3)
Frequency of drinking spirits	Never drinks spirits	132	(7.7)
(Missing = 2)	A few times per year	370	(21.7)
	1–3 times per month	667	(39.1)
	1–2 times/week	427	(25.0)
	3–4 times/week	65	(3.8)
	Almost daily	36	(2.1)
	Daily	6	(0.4)
Mean usual volume of ethanol per occasion from spirits in spirit drinkers (mls of ethanol)(Missing = 6)		118.2	(63.5)
Mean maximum volume of ethanol per occasion from spirits in spirit drinkers (mls of ethanol) (Missing = 23)		188.9	(84.3)
Drinks non-beverage alcohol	No	1582	(92.8)
	Yes	123	(7.2)
Frequency of hangover	Never	885	(51.9)
(Missing = 17)	Less than once a month	387	(22.7)
	Once a month	243	(14.3)
	Several times a month	90	(5.3)
	Once a week	46	(2.7)
	Several times a week	26	(1.5)
	Everyday	11	(0.7)
Frequency of excessive drunkenness	Never	886	(52.0)
(Missing = 17)	Less than once a month	450	(26.4)
	Once a month	227	(13.3)
	Several times a month	55	(3.2)
	Once a week	45	(2.6)
	Several times a week	16	(0.9)
	Everyday	9	(0.5)
Frequency of sleeping in clothes because of drunkenness	Never	1417	(83.1)
(Missing = 8)	Less than once a month	153	(9.0)
	Once a month	74	(4.3)
	Several times a month	23	(1.4)
	Once a week	15	(0.9)
	Several times a week	11	(0.7)
	Everyday	4	(0.2)
Frequency of failing to fulfil family or personal obligationsbecause of drinking alcohol	Never	1357	(79.6)
because of drinking	Less than once a month	141	(8.3)
(Missing = 42)	Once a month	99	(5.8)
	Several times a month	34	(2.0)
	Once a week	14	(0.8)
	Several times a week	12	(0.7)
	Everyday	6	(0.4)
Went on zapoi in the past year	No	1570	(92.1)
(Missing = 4)	Yes	131	(7.7)
Drinks large quantities of spirits without also eating some	Never/Rarely	1449	(85.0)
food	Sometimes	235	(13.8)
(Missing = 1)	Always	20	(1.2)
Ever drinks alone	Never	888	(52.1)
(Missing = 1)	Sometimes	727	(42.6)
	Often	89	(5.2)
Ever drinks before noon	Never	1177	(69.0)
(Missing = 2)	Occasionally	501	(29.4)
	Frequently	25	(1.5)
Total		1705	(100)

Analyses were carried out in Stata 11 (StataCorp, Texas)[34] and Mplus 5 (Muthén & Muthén, Los Angeles)[33].

### Ethics Statement

The Izhevsk Family Study 1 was approved by the Ethics committees of the London School of Hygiene & Tropical Medicine and the Izhevsk Medical Academy. Verbal consent was obtained from all participants, documented by interviewers on the cover page of the questionnaire before proceeding and entered into the database. Verbal consent was obtained rather than written consent due to awareness of local cultural issues concerning fear of signing official documents. This method of consent was approved by the Ethics committees of the London School of Hygiene & Tropical Medicine and the Izhevsk Medical Academy.

## Results

Of the 1,941 men interviewed in 2003–6, 1,705 (87.8%) reported that they had consumed alcohol in the past year. The majority of these men (83.4%) were in regular paid employment. Drinkers were more likely to have a higher level of education than non-drinkers (15.7% non-drinkers had higher education vs 23.4% drinkers, P = 0.007). Among drinkers the distribution by educational level was: 89 men (5.2%) had incomplete secondary level education or lower, 1,217 (71.4%) men had a secondary level education and 399 (23.4%) had a higher or incomplete higher level of education. The distribution of the sample by alcohol consumption variables and acute dysfunctional behaviours is shown in Table 1.

Missingness due to item non-response on alcohol intake and acute alcohol-related dysfunction was found to be closely associated with answers to other questions on alcohol use at the same survey. For example the question with the largest amount of missing data was frequency of failing to fulfil family or personal obligations due to drinking (missing for 42 men).This variable was more likely to be missing in men who reported more frequently sleeping in their clothes because of drunkenness (*P*<0.001). Restricting the analyses to men with complete data did not alter the results.

### Intake of beverage alcohol (latent variables)

**Figure 2:**
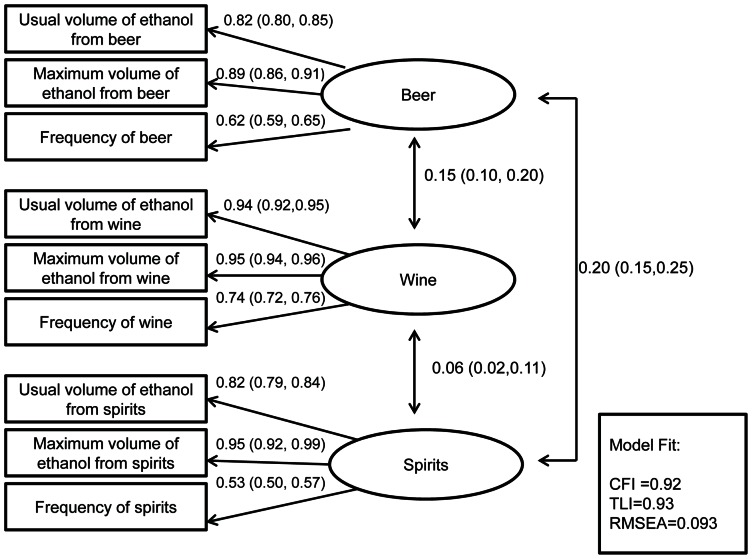
Measurement Model of Beverage Alcohol Intake with Standardized Factor Loadings (95% Confidence Intervals) for 1,705 Drinkers in the Izhevsk Family Study 1.

The measurement model used to deal with measurement error in beverage alcohol intake is shown in Figure 2. For each beverage type (beer, wine and spirits) the highest factor loading was seen for the maximum volume of ethanol consumed on one drinking occasion.

### Routine acute alcohol-related dysfunction (latent variable)

**Figure 3:**
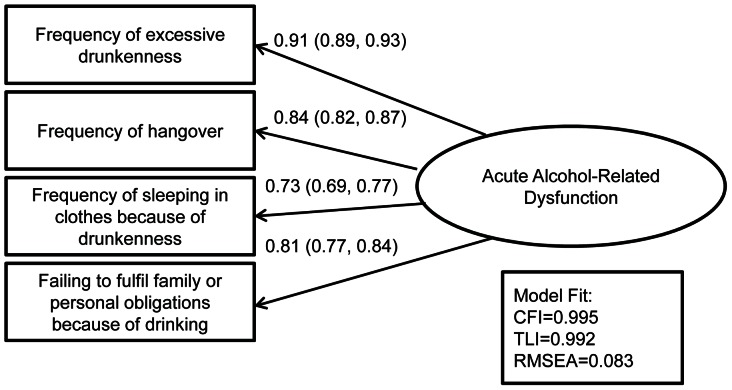
Measurement Model of Acute Alcohol-Related Dysfunction with Standardized Factor Loadings (95% Confidence Intervals) for 1,705 Drinkers in the Izhevsk Family Study 1.

The measurement model used to define acute alcohol-related dysfunction is shown in Figure 3 with factor loadings and model fit indices. All four manifest variables were strongly associated with the underlying latent factor.

### Association of age with types of alcohol consumed and acute alcohol-related dysfunction

The effect of age adjusted for education on the latent factors of beverage alcohol and acute alcohol-related dysfunction is shown in Table 2. There was strong evidence that beer intake and weak evidence that wine intake decreased with age whereas spirit consumption was not associated with age. Conversely older men were more likely to drink non-beverage alcohol (odds ratio for drinking non-beverage alcohol 1.17 per 5 year increase in age (95% CI 1.03, 1.33) although this was greatly reduced by adjusting for education (adjusted odds ratio 1.12 (95% CI 0.99, 1.28). There was strong evidence that routine acute alcohol-related dysfunction decreased with age (Table 2) but there was no evidence of an association between age and zapoi (adjusted odds ratio for one or more episodes of zapoi in the past year 0.95 per 5 year increase in age (95% CI 0.85, 1.07). Adjusting for education did not substantially change the estimates of the effects of age on either beverage alcohol intake or routine dysfunction.

**Table 2:** Relationship between Age and Latent Factors of Beer Intake, Wine Intake, Spirit Intake, and Routine Alcohol-related dysfunction among 1,705 Drinkers in the Izhevsk Family Study 1.

Latent alcohol variables at IFS-1	Unadjusted coefficient^a^	95% CI	Coefficient^a^ adjusted for education	95% CI
Beer intake	−0.20	−0.23, −0.17	−0.20	−0.24, −0.17
Wine intake	−0.04	−0.07, −0.003	−0.03	−0.07, 0.0001
Spirit intake	−0.02	−0.05, 0.02	−0.02	−0.05, 0.01
Routine alcohol-related dysfunction	−0.08	−0.11, −0.04	−0.09	−0.13, −0.05

a Coefficients represent standard deviation (SD) change in latent factor per 5 year increase in age.

### Aim 1: Associations of alcohol intake and drinking patterns with acute alcohol-related dysfunction

The estimated associations between alcohol intake and drinking patterns and the latent factor of routine acute alcohol-related dysfunction are shown in Table 3. Intake of beer, wine and spirits summarised by their respective latent variables were associated with acute dysfunction after mutual adjustment for the other drinking variables although spirit intake showed a stronger association than intake of beer or wine. The data did not hold enough information to fit a more complex specification of the model with quadratic terms for the latent alcohol intake variables therefore we were unable to formally test for non-linearity between these variables and routine acute dysfunction. Non-beverage alcohol use was strongly associated even after controlling for intake of all three types of beverage alcohol and drinking patterns, as were drinking spirits without eating and drinking before noon although drinking alone no longer maintained significance. Mutually adjusting for all the drinking variables resulted in substantial attenuation of the estimated coefficients highlighting that the drinking variables are highly correlated. Model fit of the fully adjusted model was very good (CFI 0.95; TLI 0.97; RMSEA 0.056).

**Table 3:** Relationship Between Latent Intake of Beer, Wine, Spirits, Non-beverage Alcohol use, and Drinking Patterns and Latent Routine Acute Alcohol-related Dysfunction among 1,705 Drinkers in the Izhevsk Family Study 1.

Predictors		Latent factor of Acute Alcohol-related Dysfunction			
		Adjusted for age		Adjusted for age and all other variables^b^	
		Coefficient^a^	95% CI	Coefficient^a^	95% CI
Drinks non-beverage alcohol		1.66	1.46, 1.85	0.97	0.74, 1.20
Beer intake (latent)		4.54	1.38, 7.70	0.16	0.08, 0.23
Wine intake (latent)		−0.30	−0.81, 0.21	0.25	0.17, 0.33
Spirit intake (latent)		1.05	0.93, 1.18	0.75	0.65, 0.85
Drinks large volumes of spirits without eating	Sometimes	1.32	1.16, 1.48	0.68	0.49, 0.87
	Always	1.93	1.47, 2.38	0.77	0.21, 1.33
Drinks alone	Sometimes	0.50	0.38, 0.61	0.11	−0.07, 0.29
	Often	0.93	0.70, 1.16	0.29	−0.01, 0.58
Drinks before noon	Occasionally	1.09	0.96, 1.22	0.51	0.36, 0.67
	Frequently	2.92	2.56, 3.28	0.91	0.49, 1.34

a Coefficients represent standard deviation (SD) change in continuous latent factor of routine acute alcohol-related dysfunction for respectively:

• Drinking non-beverage alcohol compared to not drinking non-beverage alcohol;

• One standard deviation increase in latent alcohol intake factors (beer, wine or spirits);

• Drinking large volume of spirits without eating “sometimes” or “always” compared to “never”;

• Drinks alone “sometimes” or “often” compared to “never”;

• Drinks before noon “occasionally” or “frequently” compared to “never”.

All estimates are adjusted for age.

b Mutually adjusted for beer intake, wine intake spirit intake, non-beverage alcohol use, spirits without food, drinking alone and drinking before noon.

The equivalent results for sporadic acute behavioural dysfunction, i.e. *zapoi*, are shown in Table 4. Again model fit of the fully adjusted model was good (CFI 0.91; TLI 0.91; RMSEA 0.075). After mutual adjustment, non-beverage alcohol use showed the strongest association out of the measures of alcohol intake while only spirit intake among the latent variables of alcohol intake maintained significance. All three drinking patterns predicted zapoi, but drinking before noon showed a particularly strong association. As with routine dysfunction, odds ratios additionally adjusted for the effect of all alcohol variables were substantially reduced compared to those adjusted only for age.

**Table 4:** Relationship Between Latent Intake of Beer, Wine, Spirits, Non-beverage Alcohol Use and Drinking Patterns and Sporadic Alcohol-related Dysfunction (Zapoi) in 1,705 Drinkers in the Izhevsk Family Study 1.

Predictors		Zapoi^a^			
		Adjusted for age		Adjusted for age and all other variables^c^	
		Odds ratio^b^	95% CI	Odds ratio^b^	95% CI
Non-beverage alcohol use		17.35	11.32, 26.59	5.96	3.43, 10.37
Beer intake (latent)		17.35	1.25, 1.87	1.24	0.98, 1.57
Wine intake (latent)		1.50	1.28, 1.75	1.00	0.83, 1.20
Spirit intake (latent)		3.03	2.39, 3.85	1.56	1.20, 2.02
Drinks large volumes of spirits without eating	Sometimes	11.38	7.68, 16.84	3.78	2.36, 6.07
	Always	25.76	10.28, 64.52	3.46	0.95, 12.58
Drinks alone	Sometimes	2.97	1.97, 4.49	1.60	0.95, 2.69
	Often	8.25	4.57, 14.91	2.25	1.02, 4.96
Drinks before noon	Occasionally	9.36	6.00, 14.60	3.84	2.26, 6.54
	Frequently	112.59	43.29, 292.83	8.61	2.72, 27.27

a Zapoi is a binary outcome.

b Odds ratios are for odds of zapoi refer to the relative odds of zapoi for respectively:

• Drinking non-beverage alcohol compared to not drinking non-beverage alcohol;

• One standard deviation increase in latent alcohol intake factors (beer, wine or spirits);

• Drinking large volumes of spirits without eating “sometimes” or “always” compared to “never”;

• Drinking alone “sometimes” or “often” compared to “never”;

• Drinking before noon “occasionally” or “frequently” compared to “never”.

All estimates are adjusted for age.

c Mutually adjusted for beer intake, wine intake, spirit intake, non-beverage alcohol use, drinking spirits without food, drinking alone and drinking before noon.

### Aim 2: Association of education with types of alcohol consumed

To aid the interpretation of the model corresponding to Figure 1 we first estimated the age adjusted associations between education and each of the latent factors of beverage alcohol intake (Table 5). Spirit intake was estimated to be lower in men with higher education compared to secondary education (coefficient −0.32, 95% CI −0.45, −0.19). In contrast there was no statistical evidence of a difference in beer or wine intake by education. There was strong evidence that non-beverage alcohol use was associated with education: odds of non-beverage alcohol use were higher in men with incomplete secondary (age adjusted odds ratio 3.06 95% CI 1.75, 5.36) and lower in men with higher education (age adjusted odds ratio 0.38 (95% CI 0.20, 0.69), relative to men with secondary level education.

**Table 5:** Relationship Between Education and Beverage Alcohol Intake Among 1,705 Drinkers in the Izhevsk Family Study 1.

Alcohol intake variable at IFS-1	Education				
	Incomplete secondary		Higher		Test for trend
	Age adjusted coefficient a	95% CI	Age adjusted coefficient a	95% CI	
Beer intake (latent)	0.08	−0.16, 0.32	−0.04	−0.17, 0.09	P = 0.40
Wine intake(latent)	0.01	−0.22, 0.24	0.10	−0.04, 0.24	P = 0.21
Spirit intake (latent)	−0.11	−0.33, 0.11	−0.32	−0.45, −0.19	P<0.001

a Coefficients for latent factor models represent standard deviation difference in latent factor compared to reference category of men in secondary education.

### Education and acute alcohol-related dysfunction

Model fit was very good when education was added to the fully adjusted models specified in Aim 1 both with (CFI 0.94; TLI 0.96; RMSEA 0.057) and without (CFI 0.93; TLI 0.96; RMSEA 0.058) a direct effect of education on routine acute alcohol-related dysfunction. However there was strong evidence that routine acute alcohol-related was lower in men with higher education even with full adjustment for alcohol intake and drinking patterns (with no evidence of a difference between men with complete and incomplete secondary education; Model 6; Table 6). Estimates are expressed as regression coefficients of the top and bottom category of education versus the middle category (secondary education) which was the most common educational level.

**Table 6:** Relationship Between Education and Latent Routine Dysfunction Adjusted for Age, and Sequentially for Alcohol Intake and Drinking Patterns in 1,705 Drinkers in the Izhevsk Family Study 1.

	Education			
	Incomplete secondary		Higher	
	Coefficient for dysfunction^a^	95% CI	Coefficient for dysfunction^a^	95% CI
Model 1: Age	0.26	−1.33, 1.85	−0.50	−0.70, −0.29
Model 2: Model 1 + beer intake wine intake and spirit intake	0.44	−0.88, 1.77	−0.42	−0.80, −0.05
Model 3: Model 1 + non-beverage alcohol use	0.09	−1.43, 1.61	−0.46	−0.62, −0.31
Model 4: Model 1 +drinking spirits without food, drinking alone and drinking before noon	0.17	−1.03, 1.37	−0.37	−0.55, −0.19
Model 5: Model 1 + drinking alone	0.28	0.03, 0.53	−0.51	−0.67, −0.36
Model 6:Fully adjusted model^b^	0.27	−0.88, 1.42	−0.35	−0.61, −0.10

a Coefficients represent standard deviation difference in continuous latent factor of acute alcohol-related dysfunction in relation to men with secondary education.

b Fully adjusted model: age + latent factor of beer intake+ latent factor of wine intake+ latent factor of spirits intake +non-beverage alcohol use+ drinking spirits without food + drinking alone + drinking before noon.

In order to interpret this result, different measures of alcohol intake and drinking patterns were sequentially removed from the model that included a direct effect of education on routine dysfunction (Table 6, Models 1–5). Changes in the estimated coefficients for the effect of education on dysfunction would highlight the pathways through which education may act.

The association between education and routine dysfunction (in particular for higher versus complete secondary education) was strongest in a model only adjusted for age (Model 1) and weakest in the fully adjusted model (Model 6). Compared to the age-adjusted model the association was reduced by additionally adjusting for intake of beverage alcohol (Model 2), non-beverage alcohol use (Model 3) or for drinking patterns (Model 4), with the exception of drinking alone (Model 5).

Unfortunately we were unable to assess interaction by education in the model due to the sparsity of the data on high levels of dysfunctional behaviours in particular among men with higher education (while regrouping of the categories would not have been satisfactory).

The same analyses were carried out for sporadic dysfunction i.e. *zapoi*. As with routine dysfunction model fit was similar with (CFI 0.90; TLI 0.89; RMSEA 0.072) and without (CFI 0.90; TLI 0.90; RMSEA 0.069) a direct effect of education but with strong evidence of a protective effect of higher education even with full adjustment for age, beverage alcohol-intake, non-beverage alcohol use and drinking patterns. The estimated direct effects of education on zapoi controlled for different measures of alcohol intake and drinking pattern are shown in Table 7.

**Table 7:** Relationship between Education and Sporadic Dysfunction (Zapoi) Adjusted for Age, and Sequentially for Alcohol Intake and Drinking Patterns in 1,705 Drinkers in the Izhevsk Family Study 1.

	Education			
	Incomplete secondary		Higher	
	Odds ratio^a^	95% CI	Odds ratio^a^	95% CI
Model 1: Age	1.57	0.83, 2.99	0.28	0.15, 0.52
Model 2: Model 1 + beer intake wine intake and spirit intake	1.65	0.80, 3.38	0.35	0.18, 0.67
Model 3: Model 1 +non-beverage alcohol use	0.91	0.34, 1.90	0.33	0.17, 0.64
Model 4: Model 1 + drinking spirits without food, drinking alone and drinking before noon	1.18	0.54, 2.58	0.44	0.22, 0.87
Model 5: Model 1 + drinking alone	1.53	0.79, 2.96	0.28	0.15, 0.54
Model 6:Fully adjusted model^b^	0.96	0.40, 2.31	0.46	0.22, 0.95

a The reference category for odds ratios is men with secondary education.

b Fully adjusted model: age + latent factor of beer intake + latent factor of wine intake + latent factor of spirit intake + non-beverage alcohol use + drinking spirits without food + drinking alone + drinking before noon.

## Discussion

In this study beverage alcohol intake, particularly spirit intake, non-beverage alcohol use, and drinking patterns (drinking spirits without eating, drinking alone and drinking before noon) were found to be strongly associated with two measures of acute alcohol-related dysfunction: a latent variable measuring routine dysfunction and *zapoi*, a measure of sporadic dysfunction. Educational level was strongly associated with both of these measures of dysfunction but in contrast to our hypothesized model this association was only partly explained by beverage alcohol intake, drinking non-beverage alcohol and two aspects of reported drinking pattern (drinking spirits without eating and drinking before noon). This is the first study to have examined these relationships within a Russian context.

Acute alcohol-related dysfunction is an important aspect of harm from alcohol. Frequency of hangover, excessive drunkenness, sleeping in clothes because of drunkenness and failing to fulfil family or personal obligations due to drinking alcohol were used as indicators of an underlying latent variable measuring routine acute alcohol-related dysfunction. All four observed variables were strong manifestations (i.e. had similarly large factor loadings) of this factor. Several previous studies have used measures of the acute consequences of alcohol consumption such as hangover and drunkenness as proxy markers of heavy drinking.[18], [21], [22] Hangover, drunkenness and passing out from alcohol have all been found to be strong predictors of more long term harm from alcohol such as self-reported general health, hospitalization and death.[19], [20] However we are not aware of any other studies which have used a principled approach to combine several markers of the acute negative consequences of alcohol consumption into one measure and then used this to identify its predictors. We have also separated routine and sporadic dysfunction and found that they had similar predictors.

Previous studies in other parts of Russia that have looked at hazardous or problematic drinking found that the prevalence of heavy drinking (≥160 g of ethanol per week), binge drinking, drinking twice a week, and mean intake of ethanol per drinking occasion were lower in more educated compared to less educated men although mean alcohol intake in the past week showed an inconsistent association with education.[15], [16] Analyses of Alcohol Use Disorders Identification Test (AUDIT) scores measured in the Izhevsk Family Study 2 (a follow-up study of men interviewed at IFS-1) found that more educated men had lower levels of alcohol-related problems but the same levels of alcohol consumption.[17]

The most intriguing aspect of our results is that in contrast to our hypothesized model the associations between education and acute alcohol-related dysfunction were only partly explained by consumption of beverage alcohol, non-beverage alcohol, and patterns of drinking such as consuming spirits without food. Given that alcohol-related dysfunction must ultimately be the result of alcohol drinking behaviour, there are only a limited number of potential explanations for these results.

The first and most obvious explanation is that our exposure and outcome measures are subject to measurement error. All information on alcohol consumption and its consequences was self-reported but it is possible that there were greater errors in the measurement of alcohol intake resulting in residual confounding. If this were the case it would suggest that measures of dysfunction provide additional information on heavy alcohol intake which is not picked up using conventional questions on frequency and volume of ethanol alone.

Aside from measurement error, it may be that we have failed to capture some important mediator-outcome confounders, some of which may be unknown, as well as other aspects of alcohol use that are correlated with education. For example there may be differences in the toxicological profile of what is consumed by educational group independent of volume or frequency of consumption. This may affect experience of dysfunction since components of alcoholic beverages in addition to ethanol have been shown to have effects on severity of hangover [27], [35]. Men in higher educational groups may consume purer sources of alcohol and therefore be less likely to experience dysfunction. Education may also be related to aspects of individual susceptibility to alcohol such as nutritional status, physical and mental health, and supportive familial and social relationships. These factors if measured may further explain the relationship between education and acute alcohol-related dysfunction.

Non-beverage alcohol use was a strong predictor of both routine and sporadic dysfunction, independent of intake of beer, wine and spirits, and drinking patterns. Future studies on alcohol consumption in Russia should include measures of non-beverage alcohol use as well as intake of beer, wine and spirits. Spirit intake was more strongly associated with acute alcohol-related dysfunction than beer or wine intake. However these results reflect levels of consumption of these beverage types amongst working-age men in Izhevsk and may not be generalisable to other populations where the proportion of ethanol consumed from spirits is lower.

There are some general limitations to the study overall. Firstly men who were living alone in 2003–2006 were excluded from the sample as data were not available for them. Also the possibility of bias induced by unknown confounders cannot be discounted. To this extent, generalizing our findings to the population as a whole has to be done with caution and suitable caveats.

In conclusion we have identified several predictors of routine and sporadic acute alcohol-related dysfunction in a sample of working-age men in Izhevsk, Russia: beverage alcohol intake particularly intake of spirits, consumption of non-beverage alcohol, drinking spirits without food, drinking alone, drinking before noon and education. The association between education and acute alcohol-related dysfunction was only partly explained by beverage alcohol intake, non-beverage alcohol consumption, drinking large quantities of spirits without food and drinking before noon. This suggests more information is needed to identify men at risk of harm from alcohol than can be identified from conventional questions on quantity and frequency of consumption. Measures of acute alcohol-related dysfunctional behaviour could be useful epidemiological tools for understanding the pathways between heavy alcohol consumption and more distal alcohol-related harm.

Finally, from a more methodological perspective, these results illustrate the challenge in formulating statistical models that convincingly identify the pathways that link educational differences to health-related behaviours and outcomes, even when the universe of potential explanatory pathways is by definition restricted, as is the case with alcohol-related dysfunction.
